# Innate immune modulation in EBV infection

**DOI:** 10.1186/2042-4280-2-1

**Published:** 2011-01-05

**Authors:** Shunbin Ning

**Affiliations:** 1 Viral Oncology Program, Sylvester Comprehensive Cancer Center; Division of Hematology/Oncology, Department of Medicine, Miller School of Medicine, University of Miami, Miami, Florida 33136, USA

## Abstract

Epstein-Barr Virus (EBV) belongs to the gammaherpesvirus family, members of which are oncogenic. Compared with other closely related herpesviruses, EBV has developed much more elaborate and sophisticated strategies for subverting host immune system, which may account for its high prevalence in immune competent hosts. Thus, study of EBV-specific immune dysregulation is important for understanding EBV latency and oncogenesis, and will identify potential molecular targets for immunotherapeutic interventions. Here I summarize the recent findings of individual EBV products in regulating host immune responses, with emphasis on the innate immune modulation.

## Introduction

Epstein-Barr Virus (EBV), known as human herpesvirus 4 (HHV4), is the first identified human cancer virus that has been shown to be associated with the development of a wide spectrum of B-cell lymphoproliferative disorders including Burkitt's lymphoma (BL), Posttransplant lymphoproliferative disorder (PTLD), and Hodgkin and non-Hodgkin lymphomas, as well as epithelial cancers including Nasopharyngeal carcinoma (NPC) and some forms of gastric carcinoma [1]. EBV is also associated with lymphomas occurring in rare patients with congenital immunodeficiency such as X-linked lymphoproliferative syndrome (XLP) [2], and plays a role in lymphoproliferative disorders which most often occur in immunocompromised patients with human immunodeficiency virus (HIV) infection (e.g. central nervous system lymphoma) or after solid organ transplantation. EBV, together with Kaposi's sarcoma-associated herpesvirus (KSHV/HHV8) and human papillomavirus (HPV), are three oncogenic viruses causally involved in acquired immune deficiency syndrome (AIDS)-associated malignancies [3].

Herpesviruses are fascinating models for scientific research as they establish lifelong persistent infections in normal immunocompetent healthy hosts as well as are able to be reactivated (replicate) for spreading to new hosts. Human herpesviruses are of particularly medical importance because they are associated with severe diseases and cancers in immunocompromised hosts [4]. Among herpesviruses, EBV is a well-established paradigm for the study of herpes viral infection, persistence, and associated malignancies [4].

## EBV infection and life cycle

EBV is spread by saliva contact, and then crosses mucosal epithelium in order to infect B cells in underlying secondary lymphoid tissues like the tonsils and adenoids. Besides spread by saliva contact, EBV may be sexually transmitted [5]. Breast milk of nursing mothers may also contain EBV which could be from an uncommon route of vertical transmission [6]. In healthy hosts, the immune system forces invading EBV to enter the destination "true latency" (latency 0) where the virus hides inside the nucleus of lymphocytes without manifesting any symptoms. The virus in "true latency" is neither pathogenic nor visible to the host immune system due to the lack of any viral protein production. But before the establishment of the final "true latency", the virus goes through several different "transitional" latency programs, named latency 3, 2 and 1, which selectively express several of the nine viral latent proteins as well as noncoding RNAs (EBERs, BARF0, and miRNAs) and are associated with EBV oncogenesis [4]. Persistent latent infection is also characterized by stable numbers of infected B cells in the blood and by the steady shedding of infectious virus into saliva. The virus is continuously surveilled by the host immune system in persistent infection [7]. However, it is invisible to the host immune system since these long-lived B cells are quiescent and express fewer proteins, and do not express any viral protein before their occasional division, during which only EBNA1, which is not recognized by cytotoxic T lymphocytes (CTLs), is expressed [8].

Besides latently infects lymphocytes and productively infects epithelial cells, EBV also infects follicular dendritic cells, mononuclear cells, plasma cells and smooth muscle cells. Infection of monocytes is likely productive [9]. However, in healthy carriers, EBV seems to be exclusively harbored in B lymphocytes [6,7]. *In vitro *infection of B cells leads to cell activation and proliferation, as well as outgrowth of transformed lymphoblastoid cell lines (LCLs, Latency 3).

Reactivation from latency in response to a specified signal requires viral genomic DNA replication and the synthesis of specific viral proteins for packing the newly replicated DNA into infectious virions. Two immediate-early (IE) transcription factors, BZLF1 and BRLF1, are responsible for expression of these packing proteins. EBV expresses a full repertoire of over 80 lytic proteins during replication period. In healthy hosts, the replication program has to be transient, rapid, and relatively rare to minimize the chances to be shut down by the host immune system. On the other hand, the virus has developed strategies to elude the immune response for successful generation of viral progeny [4,7].

The host immune system plays pivotal roles in both lytic and latent infections. It is currently deemed that a delicate modulation between host immune system, tightly controlled gene expression during distinct viral latency programs, and limited replication, enables EBV to persist in immunocompetent hosts without doing much harm [7,10]. Thus, study of the interaction between the host immune system and EBV is critical for understanding how EBV controls the balance between immune responses, undesired proliferation, and cell death, for its oncogenic benefits, and will provide a basis for potential immunotherapy for EBV-associated malignancies. Dysregulation of EBV-specific immune responses is also characteristic of EBV-associated autoimmune diseases such as rheumatoid arthritis (RA) and systemic lupus erythematosus (SLE). CTL response to EBV infection has been well documented since the discovery of EBV [11]. However, significant progresses in characterizing individual viral proteins involved in evasion of the T cell-mediated adaptive immune response have only been made in the last decade [12-16]. For example, the functional homologue of human IL10, BCRF1, elicits CD8^+ ^T cell responses, and can be processed and presented to CD8^+ ^CTLs through a TAP-independent pathway [17]. On the other hand, how EBV regulates the host innate immune system is much less understood, and only limited studies on this important subject in EBV biology have been reported recently. Here I summarize these findings which have identified individual EBV products (including proteins, noncoding RNAs, and EBV genomic DNA) involved in regulating the host immune responses in both lytic and latent infections, with focus on their roles in innate immune modulation.

## Elicitation of innate immune responses

In immunocompetent hosts, the attachment or entry of herpesviruses elicits a vigorous CD8^+ ^T cell-mediated adaptive immune response against infected cells [10]. In the meantime, like other viruses, at the early stage of infection, herpes viral infection mounts innate immune responses in the host cell, significantly manifested by activation of signaling pathways mediated by Toll-like receptors (TLRs) [18-20].

### GP350

**Figure 1:**
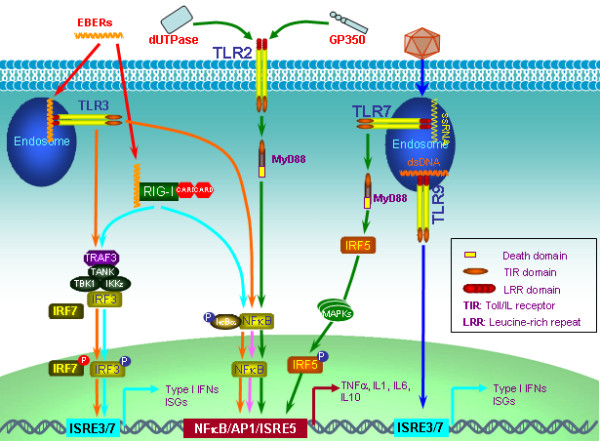
**Elicitation of innate immune response in EBV infection**. EBV dUTPase and GP350 act as ligands for TLR2. EBV EBERs can mount innate immune responses via both TLR3 and RIG-I signaling pathways. Infection of EBV also activates TLR9 signaling leading to IFNα production in pDCs. In latency, EBV manipulates the TLR7/IRF5 signaling pathway, which promotes cell proliferation. EBV products are indicated in red fonts.

Numerous studies with EBV GP350 (encoded by BLLF1), the major envelope glycoprotein which mediates EBV entry into B cells through interaction with its B-cell receptor CD21, have shown that acute and chronic EBV infections *in vitro *result in changes in secretion of TNF-α, IL-1β, IL-6, and IL-10 [21]. Like other herpesviruses such as herpes simplex virus (HSV) [22], varicella zoster virus (VZV) [19] and cytomegalovirus (CMV) [23], intact EBV virions can be recognized by TLR2 in epithelial cells and monocytes, and this process is likely mediated by GP350, suggesting that GP350 may act as a ligand for TLR2 [24] (Figure 1).

### EBV dUTPase

In addition to the envelope protein GP350, the nonstructural protein dUTPase, which is encoded by the gene BLLF3 and is one of the early antigens (EA), is also a ligand recognized by TLR2 [25]. Recognition of EBV dUTPase by TLR2 activates NFκkappaB via a MyD88-dependent signaling cascade, and induces expression of proinflammatory cytokines in macrophages [25] (Figure 1). EBV LF1 (ORF10) and LF2 (ORF11) both contain a dUTPase-like domain. However, LF2 dysregulation of host immune response may be independent of its dUTPase-like domain [26].

### EBERS

In latent infection, EBV constitutively encodes two noncoding and nonpolyadenylated small nuclear RNAs, EBV-encoded small nuclear RNA 1 (EBER1) and EBER2, which form dsRNA-like stem-loop structure by intermolecular base-pairing. EBERs have extensive structural similarity to adenoviruses VA1 and VA2 as well as cellular U6 small RNAs. EBERs are transcribed by RNA polymerase III and therefore characterized by a 5'-triphosphate moiety [27]. These features (dsRNA-like structure and 5'-PPP) render EBERs capable to function as ligands for the intracellular RNA receptor, Retinoic acid-inducible gene I (RIG-I). Recognition of EBERs by RIG-I activates interferon regulatory factor 3 (IRF3) and induces interferons (IFNs), IFN-stimulated genes (ISGs) [28], and the anti-inflammatory cytokine IL10 [29] (Figure 1). EBERs especially EBER1 can also be released by secretion of the cellular partner, La (systemic lupus erythematosus-associated antigen), from EBV-infected cells and then lead to immune activation through recognition by TLR3 and induce type I IFNs and inflammatory cytokines [30] (Figure 1). In addition to RIG-I and TLR3, EBERs also bind to IFN-inducible dsRNA-dependent protein kinase (PKR), and inhibit PKR phosphorylation and mediated apoptosis [31] (Figure 1).

Type I IFNs and proinflammatory cytokines induced by EBER1 are released from EBV-infected cells [30]. Thus, EBER1 may play a role in immunopathologic diseases associated with acute EBV infection such as infectious mononucleosis (IM), chronic active EBV infection, and EBV-associated hemophagocytic lymphohistiocytosis, as well as in EBV-associated autoimmune diseases such as SLE.

In addition to EBERs, EBV latency expresses a CG-rich transcript, IR4, with dsRNA-like structure. IR4 induces type I IFNs through unclear mechanism [32].

### EBV and TLR signaling

Interaction between viruses and TLR signaling plays a pivotal role in virus-mediated innate immune elicitation and evasion. As stated above, EBV GP350, dUTPase, and EBERs can orchestrate TLR-mediated innate immune responses. Furthermore, increasing evidence has disclosed the interaction between EBV and TLR signaling with distinct outcomes, depending on cell types. In B lymphocytes, primary infection of EBV induces expression of TLR7 and downregulates expression of TLR9, as well as activates TLR7 signaling leading to expression of the downstream target IRF5 and cell proliferation [33] (Figure 1). Controversially, another study has reported that primary infection of EBV impairs the effect of TLR7/8/9 stimulation on B cell proliferation [34]. In plasmacytoid dendritic cells, infection of EBV activates TLR9 signaling pathway leading to IFNα production, and promotes activation of NK cells and IFNγ-producing CD3+ T cells [35] (Figure 1). Since TLR9 recognizes CpG DNA motifs from bacterial or viral genomes, presuming that EBV genomic DNA may service as TLR9 ligand. In monocytes, both TLR9 and TLR2 contribute to immune responses elicited by EBV infection [24,36]. As such, the murid gammaherpesvirus 68 (MHV68), which is an animal model for study of human gammaherpesviruses, also activates antiviral immune responses in dendritic cells through TLR9 signaling pathway [37]. However, primary infection of KSHV, another gammaherpesvirus, results in TLR3-dependent induction of proinflammatory chemokines and IFN, most notably CXCL10 and IFNβ in monocytes [38]. Interestingly, TLR7/8 stimulation also reactivates KSHV from latency [38].

### EBV and autoimmune diseases

EBV has been implicated in autoimmune diseases including multiple sclerosis [39], RA [40] and SLE [41], underscored by the fact that EBNA1 was initially identified as the target antigen of sera from RA patients [42]. EBNA1 is expressed in all types of latency as well as lytic infection. Three fragments of EBNA1 protein, including 398-PPPGMRPP-404, 35-GPAGPRGGGRGRGRGRGRGHNDGG-58, and 58-GGSGSGPRHRDGVRR-72 mimics the self-antigens Sm B/B', Sm D1, and Ro, respectively, and therefore EBNA1 is believed to play a potential role in SLE [43]. Similarly, EBNA2 amino acids 354-GRGKGKSRDKQRKPGGPWRP-373 mimics Sm D1 antigen and may also contribute to SLE [43].

In addition to EBNA1 and EBNA2, EBERs exist in a snRNP complex containing La antigen specific to SLE and Sjogren syndrome, and therefore are recognized specifically by La antibody [44]. Given that recent studies showing the ability of EBER1 to provoke immune responses [28,30,31], it is very intriguing to study if EBER1 play a role in SLE in certain population.

Considering that TLRs, including TLR7, -8, and -9, are also implicated in autoimmune diseases [45,46], these TLRs may also contribute to EBV-associated autoimmune diseases, since EBV is able to regulate their expression [33,34] and may have some other interactions with signaling pathways mediated by these TLRs.

## Evasion of innate immune responses

Most viruses have evolved to encode strategies to elude host immune responses for successful replication in the host cell [47-53]. The fact that EBV infects and establishes life-long persistence in more than 95% of the adult population indicates that it is very successful in subverting host immune surveillance. In fact, EBV encodes more ingenious tricks such as invoking the host ubiquitination-proteasome system, compared with its close member in the gamma herpes family, KSHV, which encodes a larger volume of products for this purpose. KSHV infects less than 2% of the general population, indicating striking differences in their prevalence and abilities to subvert host immune surveillance. Thus, EBV has been a paradigm for studying host-virus interactions. However, despite this fact and its medical importance, EBV has been poorly understood in terms of its evasion of host innate immunity. From limited findings reported in recent years, three main strategies can be identified for this purpose: (1) manipulating type I and II IFN Jak-STAT signaling pathways; (2) regulating expression and activity of IRFs; and (3) repressing apoptosis signaling pathways.

### Immune evasion in latency

Limiting the number of expressed viral proteins and the levels of each expressed viral protein are likely two main strategies for EBV to escape the host immune system in latency [13]. EBV only expresses limited proteins in latency (9 in latency 3 and less in latency 1 and 2), and both EBNA1 and EBNA3C have been shown to be able to limit their own levels. In order to perform normal latency functions with these limited proteins, EBV encodes additional non-translated small RNAs (EBERs and miRNAs), which can not be detected by T cells looking for small peptides presented on MHC molecules [13]. In addition, memory B cells, the potential site for EBV long-term persistence [54], are not visible to immune system [8].

#### EBNA1

**Figure 2:**
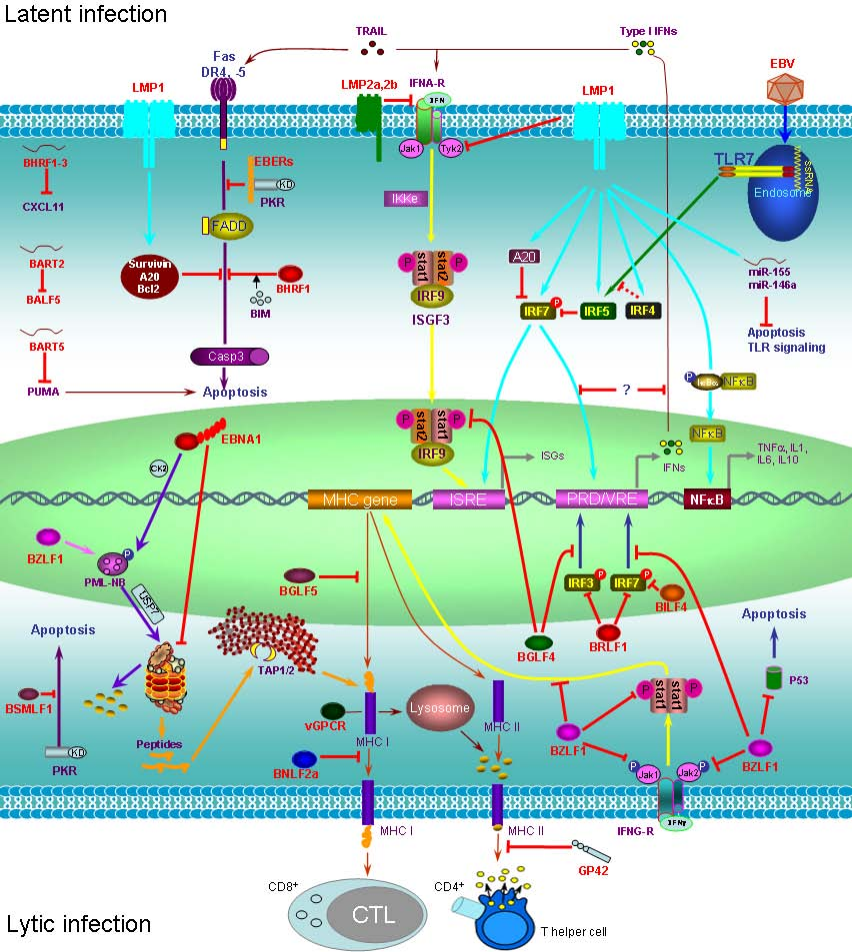
**Evasion of immune responses by individual EBV products**. Individual EBV products, including proteins, miRNAs, and EBERs, are shown to evade immune responses in both latent (upper) and lytic (lower) infections. For innate immune evasion, three main strategies are employed: (1) manipulating type I and II IFN Jak-STAT pathway; (2) regulating expression and activity of IRFs, and (3) repressing apoptosis signaling. EBV products are indicated in red fonts. PRD: positive regulatory domain; VRE: virus responsive element; ISRE: IFN-stimulated response element

EBV EBNA1 is the only EBV protein expressed in all latency program and lytic cycle, and is essential for persistence of EBV genome and establishment of latency in the host cell. EBNA1 is a typical example that escapes proteasomal processing, a process for the generation of peptides for the MHC class I antigen presentation. The long Gly-Ala repeats of EBNA1 are necessary for its escape of proteasomal processing through possible interference with the recognition and unfolding functions of the 19 S subunit [3,55] (Figure 2). The KSHV functional homolog of EBNA1, latency-associated nuclear antigen (LANA), also blocks proteasomal degradation and inhibits MHC class I-mediated antigen presentation [56]. Furthermore, EBNA1 prevents its own synthesis to keep at low but sustainable levels to avoid recognition by CTL [57]. Paradoxically, EBNA1 contains 3 peptide fragments which cross-react with three autoantigens and therefore may contribute to development of autoimmune disorders SLE, RA and multiple sclerosis [43].

EBNA1 recruits the host CK2 kinase to phosphorylate promyelocytic leukaemia nuclear protein (PML, also known as TRIM19 and RNF71) nuclear bodies (PML-NBs), and this phosphorylation leads to PML disruption through the ubiquitination pathway mediated by the ubiquitin-specific protease 7 (USP7, also known as herpesvirus-associated ubiquitin-specific protease (HAUSP)) in NPC cell lines [58,59] (Figure 2). PML is an ISG which is a multiple functional protein with an important role in antiviral responses [60].

Recent studies have shown that EBNA1, in addition to EBNA3C and LMP1, promotes genomic instability and reactive oxygen species (ROS)-mediated DNA damage response [61,62], and may facilitate c-myc translocation. These findings argue that EBNA1 is a potential oncogene [63].

#### LMP1

Latent membrane protein 1 (LMP1) is accepted as the principal EBV oncoprotein which promotes cell growth and transformation in multiple cell backgrounds and in transgenic mice. As a member of the tumor necrosis factor receptor (TNFR) superfamily, LMP1 shares many signaling intermediates with TLRs, and both LMP1 and TLRs activate NFκkappaB, a subset of IRFs, and AP1. Furthermore, LMP1 is a pleiotropic factor with distinct functions including metastasis, apoptotic resistance, and immune modulation, which heavily depend on its proper intracellular trafficking and the host cellular context [64]. It has been shown that at least in human embryonic kidney (HEK) cells, LMP1 principally signals from intracellular compartments [65]. The immune modulation function of LMP1 may associate with exosome which plays a role in antigen transfer. In fact, LMP1 is enriched in exosomes isolated from EBV-positive B cells and epithelial cells, and may stimulate biogenesis and secretion of exosomes in these cells [64]. In EBV-transformed B lymphocytes, a small portion of LMP1 undergoes phosphorylation, and phosphorylated LMP1 is preferentially associated with vimentin in the cytoskeleton network [66].

LMP1 N-terminal transmembrane domain interacts with Tyk2 and consequently, suppresses phosphorylation of both STAT1 and -2 and subsequently blocks type I IFN-mediated antiviral responses [67]. Paradoxically, LMP1 has also been shown to stimulate tyrosine phosphorylation of STAT1 and induces its expression in EBV-transformed B cells [68,69]. LMP1 was also shown to have antiviral effect by inducing type I IFNs upon superinfection through activation of NFκkappaB and IRF7 by its C-terminal activation regions (CTARs) [70] and by inducing some ISGs such as STAT1, 2',5'-oligoadenylate-synthetases (OAS) and ISG15 [71]. Furthermore, LMP1 induces a larger spectrum of genes involved in immune regulation such as chemokines CCL17 and CCL22, interleukins IL10, IL8 and IL6, and antigen processing and presentation proteins MHC class I and II and TAP2 (Figure 2). Moreover, LMP1 expressed at high levels induces autophagy [72], a cellular process which enhances antigen presentation to expose the infected cells to immune system. Thus, LMP1 seems to have opposite and diverse functions in immune modulation. In line with its immune repression function, an evolutionarily well-conserved immunosuppressive domain has been identified in its first transmembrane helix located at the LALLFWL sequence [64]. Regulation of type I Jak-STAT IFN pathway by LMP1 may represent an evolved strategy for EBV to balance the complexity of EBV/host interaction for its long-term persistence.

Apoptosis plays an important role in viral innate immune responses [73]. LMP1 is a well known anti-apoptotic protein which induces some anti-apoptotic proteins such as Survivin, A20, and Bcl-2 (Figure 2).

Besides its immune modulation role in latency, LMP1 is upregulated in hairy leukoplakia, the only pathologic manifestation of permissive EBV replication, possibly providing survival and immune evasion signals [74].

For a comprehensive understanding of the immune modulation functions of LMP1, see the excellent review [64].

#### EBERs

As stated above, EBERs can orchestrate immune responses mediated by TLR3 or RIG-I and induces inflammatory cytokines and type I IFNs. EBERs-induced type I IFNs can trigger apoptosis in EBV-infected cells [31], and helps the infected cells prevent from superinfection. But EBERs do not confer IFN resistance [75]. EBV has developed strategies to counteract this IFN-induced apoptosis. First, EBERs themselves binds to PKR *in vitro*, a key mediator of the antiviral effect of IFNs, and inhibit PKR- or IFNα-mediated apoptosis [31] (Figure 2), although in BL cells, EBER inhibition of PKR-mediated apoptosis is likely not accomplished by direct inhibition of PKR [76]. Second, BHRF1, a homolog of cellular Bcl-2, blocks apoptosis by binding to a limited amount of Bim [77] (Figure 2). Third, as stated above, LMP1 induces expression of anti-apoptosis proteins. In addition, EBNA2 is resistant to IFN-induced anti-proliferation by reducing or abolishing expression of selected ISGs including ISG54, PKR, OAS, and IFI6-16 [78].

#### EBV-encoded miRNAs

EBV encodes at least 25 miRNAs [79]. These miRNAs are encoded by two transcripts, one set in intronic regions of the BamH I-A rightward transcript (BART) gene and the other set maps to the 5'-UTR (BHRF1-1 miRNA) and 3-UTR (BHRF1-2, 1-3 miRNAs) of the BHRF gene. The three BHRF1 miRNAs are expressed during type 3 latency while the large cluster of BART miRNAs (22 miRNAs) are expressed during type 2 latency [80]. With the exception of miR-BART2, all of the BART-derived miRNAs are mapped to two clusters. BART2 miRNA is highly expressed in primary BL (type 1) and primary effusion lymphomas (PEL). The functions of these miRNAs are largely unknown [81,82], but recently their important roles in EBV pathogenesis and oncogenesis have been increasingly recognized. BART5 miRNA targets PUMA, a pro-apoptotic factor of the Bcl2 family, and therefore promotes host cell survival [83]. BART2 miRNA targets the EBV DNA polymerase BALF5 for degradation, and therefore effectively inhibits lytic replication [84]. BHRF1-3 miRNA represses expression of CXCL11 [81].

#### EBV-induced host miRNAs

miR-155 is produced from B cell intergration cluster (BIC) transcript, and is a novel crucial regulator of innate immunity, and it is also an important oncogenic miRNA (oncomiR) that is implicated in various lymphoid malignancies. miR-155 targets SHIP1 [85], IKKε [86], Table 2 [87], SOCS1 [88], and MyD88 [89], all of which are important intermediates of innate immune signaling pathways. SHIP1, IKKε, and SOCS1 also play roles in cancers. We have evidence showing that miR-155 also targets SHIP1 in the EBV context (data to be published). In addition, miR-155 targets FOXO3a, and therefore plays an important role in breast cancers [90]. Unlike KSHV which encodes a viral ortholog of miR-155 [91,92], EBV does not encode miR-155 ortholog but induces expression of cellular miR-155 by LMP1 signaling through NFκkappaB and AP1 [93-95]. We have evidence showing that BIC transcript is also induced by IRF4 in EBV latency as well as in human T-cell leukemia virus 1 (HTLV1)-infected cells, and the levels of BIC and IRF4 are correlated in HTLV1-associated adult T cell leukemia/lymphoma (ATLL) tumors (data to be published).

In addition, EBV induces miR-146, miR-21, miR-23a, miR-24, miR-27a, and miR-34a in its latency [96-98]. Like miR-155, both miR-146 and miR-21 are also oncogenic and are important regulators of innate immune responses. miR-146 targets TRAF6, IRAK1 and -2, and therefore attenuates type I IFN production in macrophages [99]. miR-21 targets the proinflammatory tumor suppressor PDCD4 and therefore promotes cell transformation and negatively regulates TLR4 signaling [100,101].

#### EBNA2

EBNA2 has resistance to type I IFNs by reducing or abolishing expression of four ISGs: ISG54, PKR, OAS, and IFI6-16 [78]. However, EBNA2 can stimulate IFNβ expression and ISGF3 activity in BL cell lines [102].

#### LMP2A and 2B

LMP2A and -2B limit IFN signaling by promoting turnover of both type I and II IFN receptors, IFNAR and IFNGR [103].

#### Evasion of IRF7-mediated IFN responses in latency

EBV type 3 latency expresses a few more proteins than other latency programs, and mainly exists in immunocompromised hosts *in vivo *and lymphoblastoid cell lines *in vitro*. This latency is associated with distinct lymphoproliferative diseases in patients infected with HIV or suffering from other immunodeficiency conditions, such as iatrogenic immunodeficiency following solid organ transplantation. Latency 3 also exists in healthy people [104], presumably as the transition process to the destination "true latency".

LMP1 is expressed at a much higher level in latency 3 compared with other latency programs. We have shown ample evidence that LMP1 in latency 3 induces as well as activates IRF7 [105-107], the "master" regulator of type I IFN responses [108]. However, activated IRF7 does not induce considerable type I IFNs in EBV latency. The mechanism underlying this paradox is unclear. Understanding how EBV escapes the IRF7/IFN signaling pathway but retains IRF7's oncogenic activity is of great interest, as their outcomes shape not only the immune response to viral infection, but also affect aspects of host cell proliferation and survival. Recently, another IRF7 splicing variant, IRF7C, which is also induced by LMP1, has been identified to inhibit IRF7 transcriptional activity by competing with IRF7 for binding to IFN promoters [109]. This may provide a valuable clue for escape of IRF7-mediated IFN signaling in EBV latency. We are performing genome-wide screening to identify regulators of the IRF7/IFN signaling in EBV latency.

### Immune evasion in lytic cycle

#### EBV GPCR

A systematic screen of EBV lytic genes has identified BILF1, the EBV G protein-coupled receptor (GPCR) homolog which has constitutive signaling functions, as a specific inhibitor for MHC class I presentation on cell surface [110]. BILF1 targets MHC class I molecules for lysosomal degradation, and therefore abrogates its recognition by immune T cells (Figure 2). However, BILF1 exerts this effect through direct interaction with MHC class I complexes, independently of its GPCR signaling, and the underlying mechanism is distinct from those of other viral proteins which target MHC class I for degradation [110]. BILF1 also constitutively inhibits PKR phosphorylation [111,112]. KSHV GPCR homolog (ORF74) does not have this function, whereas the BILF1 homolog of the Rhesus γ_1_-herpesvirus CeHV15 has similar function with EBV BILF1 in downregulation of MHC class I [110].

#### BGLF5 and BNLF2a

Two other EBV proteins have been described which significantly suppress adaptive immune responses. BGLF5, the EBV alkaline exonuclease (DNase), helps EBV to escape host T-cell recognition and elimination of the infected cell by shutting off the expression of MHC class I and II genes [113]. BNLF2a, an EBV lytic cycle early protein, blocks MHC class I presentation through inactivation of the TAP1/TAP2 peptide transporter to impair CD8^+ ^T-cell response [114,115] (Figure 2).

#### BZLF1 and BRLF1

The IE transcription factor BZLF1 is homologous to HSV1 ICP0 and host AP1. Like HSV1 ICP0 and EBV EBNA1, BZLF1 also disrupts PML [116]. BZLF1 specifically inhibits IFNγ signaling at multiple levels: BZLF1 inhibits IFNγ-stimulated STAT1 Tyr701 phosphorylation as well as tyrosine phosphorylation of Jak1 and Jak2, decreases expression of IFNγRα, and reduces IFNγ-induced MHC II expression [117]. BZLF1 interacts with P53 and inhibits transcription of both proteins. BZLF1 also targets P53 protein for degradation through MDM2-independent ubiquitination pathway [118] (Figure 2). In addition, as a transcription factor, BZLF1 inhibits TNFR1 expression through direct interaction with C/EBP proteins [119].

We have shown that BZLF1 inhibits IRF7 transcriptional activity [120]. BZLF1 and IRF7 physically interact. But BZLF1 had no effect on IRF7 nuclear translocation. The exact mechanism remains further study.

The other IE protein, BRLF1, decreases expression of IRF3 and -7, and therefore negatively regulates IFN responses to facilitate viral replication [121] (Figure 2).

#### BGLF4

BGLF4, the only EBV protein kinase (PK) whose ortholog UL13 in HSV1 has been implicated in counteracting IFN production [122], was identified as an IRF3-interacting protein in yeast two-hybrid screening [123]. BGLF4 phosphorylates IRF3 *in vitro *and does not prevent IRF3 dimerization and nuclear translocation. Phosphorylation of IRF3 by BGLF4 did not result in its proteasomal degradation, instead, diminished IRF3 binding to DNA, probably through affecting IRF3 optimal conformation for stable DNA binding [123] (Figure 2). BGLF4 also inhibits STAT1 Tyr701 phosphorylation [123] (Figure 2).

#### BILF4

Screening of EBV ORF library has identified BILF4 (LF2), which is also expressed at IE stage, as a potent inhibitor for IRF7-stimulated IFN promoter activity [26]. This inhibition effect is specific to IRF7 but not to IRF3. BILF4 is located in the nucleus and does not inhibit IRF7 phosphorylation and nuclear translocation; instead, it interrupts IRF7 dimerization through interaction with IRF7 central IRF association domain (IAD) [26]. Rhesus lymphocryptovirus LF ortholog has similar function with EBV LF2 [26]. LF2 is not necessary for EBV replication; in fact, it inhibits EBV replication [124].

#### BARF1

EBV-encoded BARF1 functions as a colony-stimulating factor 1 (CSF1) receptor for human CSF1, which is known to induce proliferation of bone marrow macrophages and promotes mononuclear cells to release cytokines such as type I IFNs, TNFα, and IL1 [125].

#### BSMLF1

BSMLF1 is known as SM, BMLF1, EB2 and Mta. BSMLF1 is a transactivator and mRNA export factor that is essential for EBV replication. BSMLF1 binds to and inhibits PKR activation, and also interacts with TAP, CRM1, and PML-NB P110b subunit [126].

#### GP42

The envelope glycoprotein, GP42 which is encoded by BZLF2, binds to MHC class II and mediates viral entry to B cells. Binding of GP42 to MHC class II subverts CD4^+ ^T cell activation through disruption of the interaction between MHC class II and T cell receptor (TCR) [14] (Figure 2).

## Perspectives

It is clear that modulation of the host innate immune responses is a key component in EBV lifecycle. EBV, compared with other herpesviruses, encodes more sophisticated and successful strategies for this purpose. EBV encodes fewer products to accomplish this goal and successfully infects more than 90% of the population. This topic had not been taken into account until recently, emerging evidence show that EBV noncoding RNAs, IE transactivators, EBNA1, and LMP1, play important roles in these processes. However, these limited pieces of evidence are far away from clear to elucidate the whole picture of how EBV subverts host innate immune system. The study of EBV-noncoding RNAs and miRNAs in modulation of innate immune responses is just beginning, and will be the focus of this topic in the near future. How innate immune signaling pathways such as TLR and RLR signaling pathways respond to EBV primary infection is not clear, although this is the key to understand how the virus establishes latency after primary infection. Whether the innate immune signaling manipulates EBV reactivation is also an interesting question. Understanding the mechanisms whereby EBV evades innate immune responses to establish long-life latency and to develop malignancies is paramount for therapeutics of EBV-associated malignancies.

## Competing interests

The author declares that he has no competing interests.

## Acknowledgements

This work is supported by the State of Florida Biomedical Research Programs (1BN-07) and NCI (1P30CA147890-01).
